# Stromal Vascular Fraction Therapy to Reduce Inflammation and Improve Cartilage Regeneration in Osteoarthritis Nude Rats

**DOI:** 10.1155/sci/5356264

**Published:** 2025-02-27

**Authors:** Xuan-Qi Zheng, Tong Wu, Minwei Zhao, Chun-Li Song

**Affiliations:** ^1^Department of Orthopaedics, Peking University Third Hospital, Beijing, China; ^2^Beijing Key Laboratory of Spinal Disease Research, Beijing, China; ^3^Engineering Research Center of Bone and Joint Precision Medicine, Ministry of Education, Beijing, China

**Keywords:** cartilage, inflammation, osteoarthritis, SVF

## Abstract

**Aims:** To evaluate the efficacy of stromal vascular fraction (SVF) in treating osteoarthritis (OA).

**Background:** OA is a common degenerative disease, the most important manifestation of which is cartilage destruction and inflammation. The SVF is a mixed group of multiple cells extracted from adipose tissue with a certain ability to promote tissue repair. However, the biological safety and efficacy of human derived SVF in treating OA have not been confirmed.

**Methods:** Seventy-six nude rats were used in this experiment. The rat OA model was constructed with anterior cruciate ligament transection (ACLT). After 4 weeks, SVF cells were injected into the joint cavity once. After 12 weeks, the experimental animals were sacrificed and decalcified sections were subjected to hematoxylin and eosin (H&E), safranine O staining, and AP-PAS staining and immunohistochemistry for inflammation markers.

**Results:** After surgery, the knee joint swells, pain intensifies, and the joint space narrows. The results of H&E, safranine O, and AP-PAS staining showed that the cartilage tissue was damaged in the ACLT-OA group and the treatment of SVF can reduce cartilage degradation. The numbers of ADAMTS-5-, MMP-13-, and IL-1*β*-positive cells significantly decreased and type II collagen-positive cells were more frequently detected in the ACLT-OA group compared with that in the control group, the treatment of SVF can reduce inflammation.

**Conclusion:** SVF cells can be safely used to treat OA and can both effectively reduce the progression of joint inflammation and promote cartilage regeneration.

## 1. Introduction

Osteoarthritis (OA) has a high incidence rate and heavy burden [1, 2]. While the etiology of OA is diverse, the main manifestations are inflammation [3] and cartilage destruction. Current treatment methods include physical therapy, drug therapy, and surgical treatment, but the short- and long-term effects are limited [4].

The stromal vascular fraction (SVF) consists of the aqueous component remaining after the removal of mature adipocytes from adipose tissue and contains a variety of cells capable of repair function as well as a mixture of cytokines [5, 6]. Adipose-derived stem cells (ADSCs) are an important component of the SVF. ADSCs have the potential for multidirectional differentiation and the ability to promote vascular regeneration and regulate the immune system [7]. Their potential in osteogenesis, bone healing [8, 9], and cartilage regeneration [10] has been fully confirmed and widely studied [11, 12]. However, compared with the effects of simple stem cells, the various cells in the SVF exert synergistic effects; their interactions with each other result in increased tissue repair and wound healing ability through autocrine and paracrine actions.

The advantages of injecting autologous SVF into the knee joint cavity to treat OA lie in its wide availability, easy access, simple operability, and good repeatability, as well as the autologous source, low associated pollution, and high safety [13, 14]. The animal experiments on the application of SVF in the treatment of OA are summarized in Table 1.

To evaluate the biological safety and efficacy of SVF in the treatment of OA, we established a model of knee OA induced by anterior cruciate ligament transection (ACLT) in nude rats [24, 25] and then, injected human derived SVF cells into the knee joint cavity of the nude rats. After 8 weeks, we evaluated the level of articular cartilage regeneration and inflammation.

## 2. Method and Materials

### 2.1. Animals and Experiments

Thirty-six 8–9-week-old male nude rats were purchased from Vital River Laboratory Animal Technology Co., Ltd., Beijing, China. The mice were kept in standard animal facilities in controlled temperatures (22°C) and photoperiod (12 h of light and 12 h of darkness) and had free access to fresh water and food. The experiment was conducted under the Guidelines for Animal Experiments of Peking University Third Hospital, which was approved by the Institutional Animal Ethics Committee (A2022022).

### 2.2. SVF Preparation

The SVF used in this study was purchased from Zhongyuanxiehe. Ltd., Co. In brief, adipose tissue is provided by patients undergoing liposuction. And after the upper layer of adipose tissue from the adipose tissue collection bottle was transferred to a centrifuge tube, physiological saline was added, and the content of the tube was washed and cleaned three times. Then, the upper layer of fat was transferred to a new 50-mL centrifuge tube and an equal volume of collagenase was added, followed by digestion for 30–60 min at 37°C and 1200 rpm. After digestion, the tube was centrifuged at 800 rpm for 3 min. Subsequently, the upper layer of fat was removed, and the digested adipose tissue was passed through a 100-μm cell filter. The filtrate was collected, the volume was brought to 45 mL using physiological saline, and the sample was centrifuged at 1500 rpm for 10 min; this process was repeated twice. After centrifugation, the supernatant was discarded and the cell pellet was resuspended in 4.5 mL of physiological saline to obtain the SVF product.

### 2.3. Establishment of OA Model in Rats by ACLT

Thirty-six 8–9-week-old male nude rats were used in this experiment. After 1 week of adaptive feeding, ACLT was performed to establish an induced OA model [24–26]. In brief, after inducing anesthesia with isoflurane (1%–1.5%), a tourniquet was used to bind the operative limb, the knee joint capsule was opened through an incision on the medial side of the patellar tendon, and microsurgical scissors were used to cut the anterior cruciate ligament; the same procedure was performed on the contralateral side. Animals in the control group underwent an operation that included arthrotomy without ACLT in both knee joints. Immediately after the operation, the drawer test was performed to verify the stability of the knee joint. After surgery, the rats were randomly divided into four groups: the sham operation group, ACLT-OA group, low-dose SVF treatment group, and high-dose SVF treatment group. Animals in each group received an SVF (for low/high-dose SVF treatment group) or phosphate-buffered saline (PBS; for ACLT-OA group) injection 6 weeks after the operation and were sacrificed 12 weeks after the operation. Then, the knee joint was dissected and evaluated histologically.

### 2.4. Swelling Assessment

The degree of knee joint swelling was measured immediately and 3, 7, and 14 days after the operation. In brief, vernier calipers were used to measure the longest diameter of the knee joint of rats in each group and the obtained data were recorded for analysis.

### 2.5. Pain Assessment

Before and 8, 10, and 12 weeks after the operation, each rat was placed into a custom-made plastic cylinder, with the hind legs extended out of the end of the cylinder. The knee joint was slowly straightened and then flexed once every 5 s for a total of five times. If the rat hissed or showed an obvious leg recoil reaction during flexion, a score of 1 was recorded; if not, a score of 0 was recorded.

### 2.6. Histological Assessment

At 12 weeks after the operation, the rats were sacrificed by excessive inhalation of carbon dioxide and the knee joints were harvested. Samples were fixed in 4% (*v*/*v*) neutral polyformaldehyde for 24 h and decalcified in neutral 10% (*v*/*v*) EDTA solution for 1 month.

After the specimens were embedded in paraffin, 5-µm-thick sagittal tissue sections were obtained. Each section was stained with hematoxylin and eosin (H&E) and safranin O (S-O) according to the manufacturer's instructions. Another group of experienced histological researchers examined the cellular composition and morphology of the cartilage in the samples under microscopy in a blinded manner using the medial femoral condyle and medial tibial plateau scoring system of the Osteoarthritis Research Society International (OARSI) [25, 27]. The severity of synovitis was graded according to a previously described scoring system (0 = 1–2 cells, 1 = 2–4 cells, 2 = 4–9 cells, and 3 = 10 or more cells). The cell density in the synovial matrix was score from 0 to 3 (0 = normal cell density, 1 = slightly increased cell density, 2 = moderately increased cell density, and 3 = significantly increased cell density). Some methods follow the description of previous studies [28].

### 2.7. Immunohistochemistry

After sections for immunohistochemistry were deparaffinized and rehydrated, endogenous peroxidase activity was blocked by 3% H_2_O_2_ for 10 min, followed by three washes with PBS. The sections were then incubated with 0.1% trypsin for 20 min and washed three times with PBS. Subsequently, the sections were incubated with 1% (*w*/*v*) goat serum albumin for 30 min at room temperature, followed by incubation with primary antibodies (IL-1*β* 1:200; COL2 1:200; MMP13 1:200; ADAMTS5 1:200) overnight at 4°C. Negative control sections were incubated with nonspecific IgG. Then, the sections were placed in the ambient environment for 1 h to return to room temperature, washed three times with PBS, and incubated with HRP-conjugated secondary antibodies for 1 h at 37°C. Six rats from each group were used for quantitative analysis; at least three sections from each specimen were observed. The rate of positive cells in each section was quantitated by observers who were blinded to the experimental groups.

### 2.8. Statistical Analysis

The experiments were performed in triplicate. The results are presented as the mean ± S.D. Statistical analyses were performed using SPSS statistical software program 20.0. Data were analyzed by one-way analysis of variance (ANOVA) followed by Tukey's test to compare data between the control and treatment groups. Nonparametric data (OARSI and synovitis scores) were analyzed by the Kruskal–Wallis H test. *p* values less than 0.05 were considered significant.

## 3. Results

### 3.1. The Establishment of ACLT Rat Model

**Figure 1:**
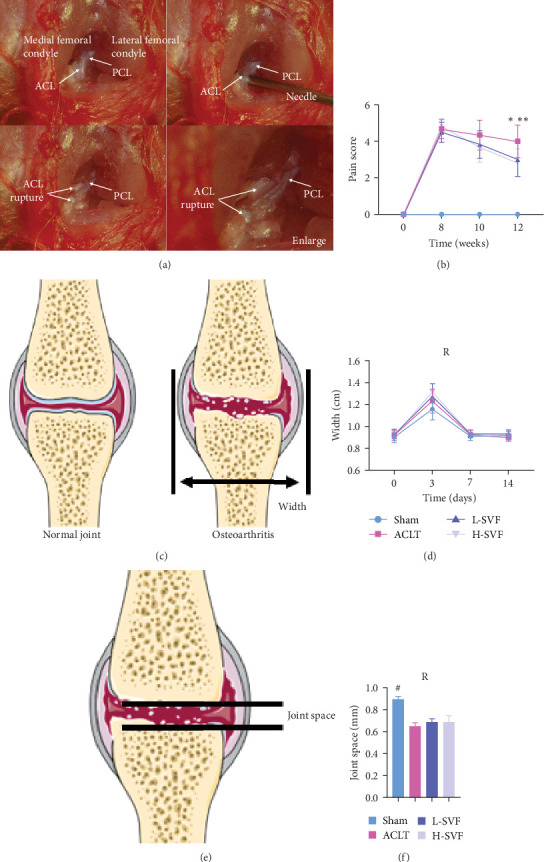
Swelling of the knee, increased pain, and narrowing of the joint space occur after surgical treatment. (A) Establishment of the experimental OA model under stereomicroscopy. (B) Score records of pain assessment after surgical treatment. (C, D) Schematic diagram and the measurement results of the measurement method of the transverse diameter of the knee joint. (E, F) Schematic diagram and the measurement results of the measurement method of the joint space of the knee joint. *⁣*^*∗∗∗*^*p* < 0.001; ^#^*p* < 0.0001.

Under stereomicroscopy, the knee joint structure of rats can be clearly observed, along with the anterior and posterior cruciate ligaments. The anterior cruciate ligament can be separated and severed using a needle, while maintaining the integrity of the posterior cruciate ligament (Figure 1A). The rats subjected to ACLT showed large joint movement and a positive drawer test result, while rats in the sham operation group showed a negative drawer test result.

### 3.2. Knee Joint Swelling After Operation

Compared with before the operation, obvious knee joint swelling was observed in rats 3 days after the operation due to the surgery and local inflammation, among other reasons. After 1 week, the swelling subsided and the size of the knee joint returned to normal, with no significant difference between the left and right knee joints. The degree of swelling was the lowest after the sham operation, but there was no significant difference between the groups (Figure 1).

### 3.3. SVF Treatment Reduce Postoperative Pain

Compared with before the operation, these rats tests positive for pain at 8 weeks after the operation, and while the pain score of the rats receiving SVF treatment decreased at 10 weeks, there was no significant difference between the groups. At 12 weeks, animals in the model group still showed high pain sensitivity, but the pain score in the SVF treatment group was significantly decreased, with no significant difference between the high- and low-dose SVF groups. The scores of the left and right limbs were similar (Figure 1B).

### 3.4. Histological Assessment

**Figure 2:**
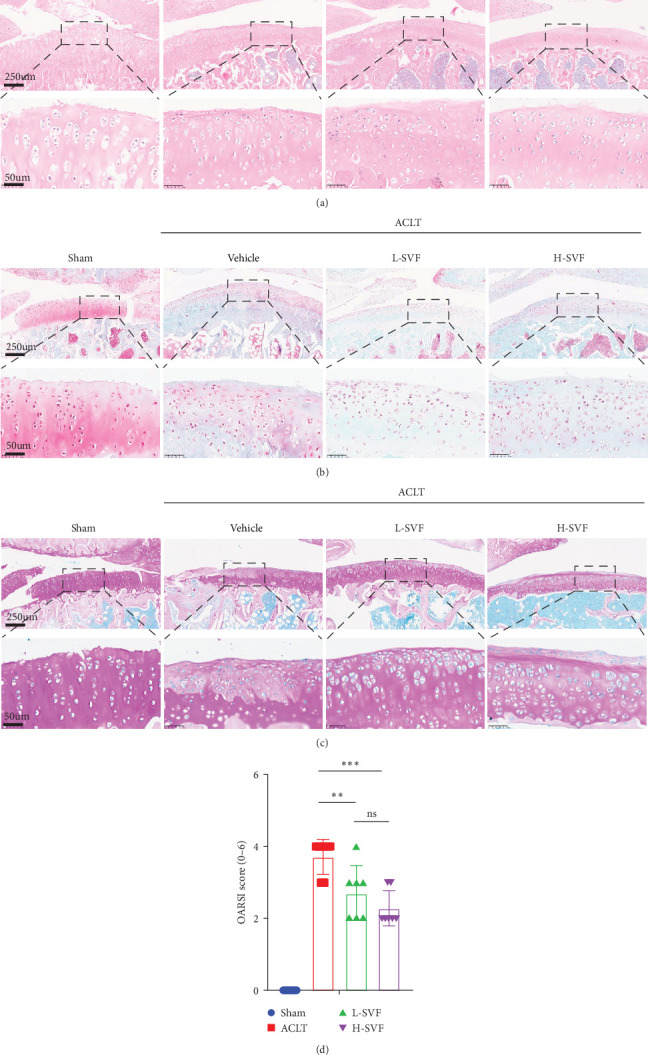
Histological assessment of osteoarthritis (OA) progression. Representative images of hematoxylin and eosin (H&E) staining (A), safranin O (S-O) staining (B), and AB-PAS staining (C). (D) The histopathological score based on the Osteoarthritis Research Society International (OARSI) OA histopathology grading system. *⁣*^*∗∗*^*p* < 0.01; *⁣*^*∗∗∗*^*p* < 0.001.

H&E staining showed that the cartilage tissue in the sham operation group had distinct layers, a normal number of chondrocytes, and a complete cartilage matrix and tidal line. In the ACLT-OA group, the cartilage tissue was damaged, the number of chondrocytes was decreased, and the cartilage matrix and tidal line were incomplete (Figure 2A).

S-O staining and AB-PAS staining showed superficial cartilage destruction, extensive proteoglycan loss, and obviously reduced cell function in the ACLT-OA group compared with the sham operation group, while less proteoglycan loss and cartilage destruction were observed in the SVF group than in the ACLT-OA group. The cartilage was evaluated according to the OARSI scoring system. The results showed the highest OARSI score in the ACLT-OA group; SVF treatment mitigated the deleterious effects on cartilage and alleviated the synovitis observed in the ACLT-OA group compared with the sham operation group. In addition, there was no statistically significant difference between the low- and high-dose SVF groups (Figure 2).

### 3.5. SVF Treatment Reduce Postoperative Inflammation

Immunohistochemical staining for IL-1 and IL-6 showed low IL-1 expression in the sham group, which increased after ACLT, indicating increased inflammation. The expression of IL-1 was slightly lower in the high- and low-dose SVF groups than in the model group, with no significant difference between the high- and low-dose SVF groups.

### 3.6. Synthesis and Degradation of Cartilage Matrix

**Figure 3:**
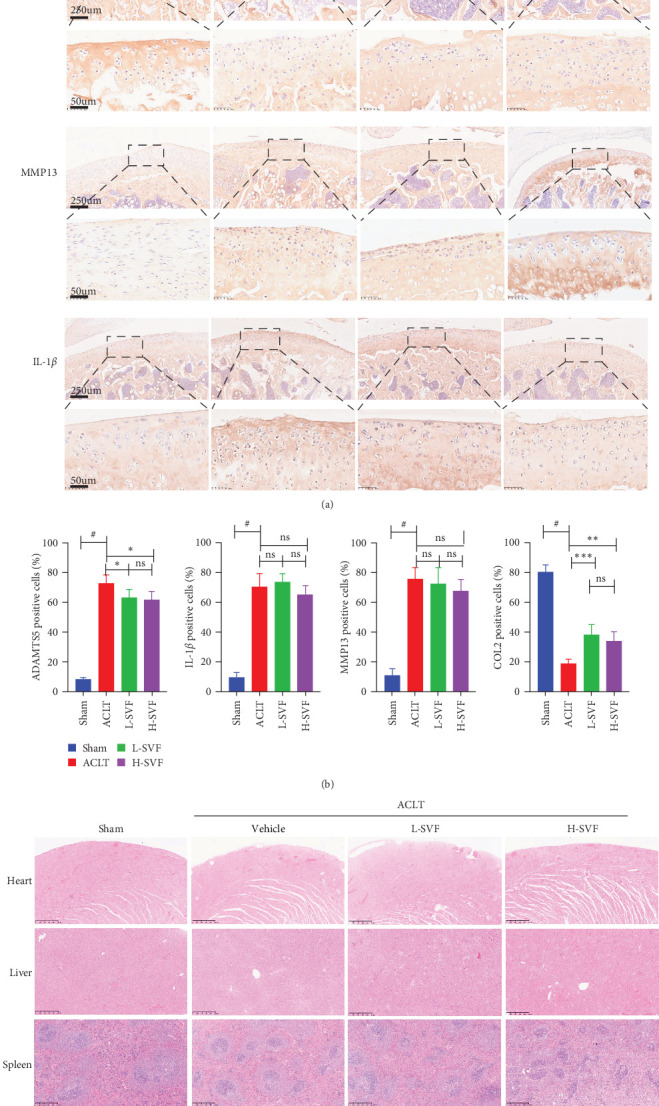
Histological assessment of cartilage regeneration, cartilage degrading enzymes, and inflammatory cytokines. (A, B) Representative images of immunohistochemical analyses of ADAMTS5, IL-1, MMP13, type II collagen. (C) Hematoxylin and eosin (H&E) staining of major organs. *⁣*^*∗*^*p* < 0.05; *⁣*^*∗∗*^*p* < 0.01; *⁣*^*∗∗∗*^*p* < 0.001; ^#^*p* < 0.0001.

The expression of ADAMTS5 was low in the sham group and increased after ACLT, and collagen degradation was accelerated. The expression of ADAMTS5 was slightly lower in the high- and low-dose SVF groups than in the model group, with no significant difference between the high- and low-dose SVF groups. MMP13 expression was low in the sham group and increased after ACLT and collagen degradation was accelerated. The expression of MMP13 was slightly lower in the high-/low-dose SVF groups than in the model group, with no significant difference between the high- and low-dose SVF groups. COL II expression was higher in the sham group and decreased after ACLT and collagen synthesis slowed. The expression of COL II was slightly higher in the high- and low-dose SVF groups than in the model group, with no significant difference between the high- and low-dose SVF groups (Figure 3A,B).

### 3.7. H&E Staining of Major Organs

H&E staining of the heart, liver, spleen, lungs, kidneys, and other organs showed no obvious damage to any of the major organs and complete structural integrity (Figure 3C).

## 4. Discussion

Cartilage destruction and inflammation are important pathophysiological mechanisms of knee OA. The emergence of stem cells has provided new ideas for the treatment of knee OA [29, 30]. In particular, SVF cells could have advantages in the treatment of OA. We found that treatment with an intra-articular injection of human derived SVF cells was safe and yielded histological results suggesting that SVF cells can effectively inhibit inflammation and reduce cartilage damage.

Mechanistic research requires reliable and stable animal models. Previous models of OA have been established in mice, rats, rabbits, pig, sheep, horses, et cetera [31, 32]. However, mice are small in size and have a complex joint anatomy. While the anatomical structure of the knee joint is clear in large animals, the unit price of large experimental animals is high, increasing the cost of research. In this experiment, we used nude rats as experimental objects.

Nude rats are similar to normal SD rats in body shape and their body weight is approximately 60%–70% of that of normal SD rats. Compared with normal SD rats, nude rats are congenitally immunodeficient and athymic, with a lack of T-cell function, normal B-cell function, and strong NK-cell activity [33–35], and these rats are generally used for tumor [36, 37] and stem cell research [38, 39].

Methods for modeling knee OA include those based on trauma, metabolism, aging, and genetics, among other methods [25, 40, 41]; however, the most common method used to rapidly induce OA is invasive intraarticular surgery. ACLT has the advantages of being simple to perform and causing minimal structural damage, and it is currently the most commonly used surgical OA modeling method [42–44]. Because ACLT mainly induces cartilage degeneration by destabilizing the joint, it induces OA lesions more quickly than meniscus resection and is, thus, easier to use in pharmaceutical research. In this experiment, the anatomical structure of the rat knee joint could be clearly observed under stereomicroscopy, and the anterior cruciate ligament of the rat could be transected under direct vision without causing other incidental injuries.

The pathogenesis of arthritis is complex, involving many biochemical, biomechanical, structural, physiological, immune, and metabolic changes. And treatments for the early and middle stages of OA have limitations. Increasing evidence from basic research and clinical trials support the potential of stem cell therapy for the treatment of OA [45–48]. As multifunctional cells, stem cells have strong potential for self-renewal, proliferation, and differentiation. At present, the mechanisms of action of stem cells include migrating to the damaged site for articular cartilage repair and reconstruction [49, 50], secreting various bioactive molecules with immune and anti-inflammatory effects, and secreting cytokines and growth factors to nourish cartilage [12, 51, 52]. These activities can reduce the inflammatory microenvironment of the diseased joint to induce the generation of new chondrocytes and alleviate the clinical symptoms of chronic pain in OA.

While the SVF dosage may have an strong impact on the results [53], in our study, no significant difference was found between the low- and high-dose SVF groups in terms of inflammation control or cartilage regeneration. Possible explanations are differences in the subjects studied and the doses administered. Thus, more evidence from laboratory and clinical studies is required to continue.

There are some limitations in this experiment. First, the cellular and molecular mechanism studies are not studied, and the dynamic changes of the inflammatory microenvironment in knee arthritis after SVF injection have not been revealed. Second, it is worth investigating the relationship between the dose administered and the dose that actually works, as well as potential ways to protect cell viability.

## 5. Conclusion

In the current study, we demonstrated that the use of human derived SVF cells in the treatment of OA in nude rats is safe. In addition, SVF treatment significantly inhibited inflammation and cartilage catabolism in rats with ACLT-induced OA. Overall, these results suggest the potential of SVF cells for use in the treatment of OA.

## Figures and Tables

**Table 1:** Summary of animal experiments on the application of stromal vascular fraction (SVF) in the treatment of osteoarthritis (OA).

Animal	OA model/operation	SVF/adipose tissue source	SVF production process	SVF dose and frequency	Duration of test observation	Ref.
66 male 12-week-old BALB/c-nu mice	Resecting the medial meniscotibial ligament under a microscope to destabilize the medial meniscus	SVF cells were extracted from the abdominal or subcutaneous fat of 10 patients who received intra-articular injection of ADRCs for the treatment of knee pain.	Celution800/CRS system (Cytori Therapeutics Inc., San Diego, CA, USA)	2.0 × 10^4^ cells/6 µl/knee	4 week and 8 week postsurgery	[15]

The NOD mouse/SCID mice	Articular cartilage destruction by fine needle 32.5 G	Adipose tissue was collected from abdominal fat tissue of 10 consenting healthy donors.	Using the extraction kits (Adistem, Australia)	200 µl containing 2 × 10^6^ SVF in platelet-rich plasma	45 days	[16]

12-week old adult male C57BL/6 mice	Severe OA in both knees by a single injection of 13 U collagenase II in 6 μl of 5 mM CaCl2	Adipose tissue from supra- or infrapatellar areas was collected from 24 patients between 50 and 80 years old indicated for complete joint substitution.	The samples were washed multiple times in phosphate-buffered saline (PBS) plus antibiotics to clean the tissue and remove residual blood. The samples were distributed within 10 g of adipose tissue per 100 mm petri dish, with a solution containing PBS, 100 units/mL penicillin and 100 μg/mL streptomycin, collagenase type IA, and dispase I. The tissue was cut into small pieces using sterile surgical scissors in a laminar flow hood and digested in a closed cell flask overnight in a shaker at 37°C, 20% O_2_, 5% CO_2_	10^5^ cells in 6 μL	1 month	[17]

New Zealand white rabbits (aged 4–6 months, weighing 3.5–4.0 kg)	Full-thickness cartilage defects extending through the cartilage layer and penetrating the subchondral bone were created surgically at the femor-opatellar groove of both hind-leg knee joints. The defects measured 4 mm in diameter and 2 mm in depth	Rabbit dorsal fat pads.	The dorsal fat pads were minced and washed with PBS, then digested using 0.075% type I collagenase in a 37C shaking water bath for 60 min. After neutralizing the collagenase, the digested tissue was filtered and centrifuged, then, the resulting pelleted SVF was resuspended and seeded in culture flasks with MSC proliferation medium	Rabbit ASCs were seeded at the density of 10^5^ per mL into the flasks and on glass coverslips into the 96-well plates, which were placed in the PHB/PHBHHx scaffolds at an appropriate initial density	10 weeks	[18]

12 skeletally mature (age: 2–3 years) female Spanish goats	A full-thickness radial incision spanning 90% of the medial meniscal width was made at the junction of the anterior and middle meniscal bodies with a No. 10 blade	Autologous SVF cells were isolated from the infrapatellar (Hoffa) fat pad (IPFP).	Rapid enzymatic digestion	2.0 × 10^6^ cells/mL in 150 mL of the mGL hydrogel.	6 months	[19]

Male C57BL/6 mice (9-weeks of age, 20–25 g)	Destabilization of the medial meniscus (DMM) mouse model	N.A. (primary hASCs were purchased from CEFO Bio Co., Ltd).	Extracellular vesicle (EV) isolation from conditioned medium of hASCs	EVs (1 × 108 particles) and PBS were given in a 6 μL volume per joint	11 week	[20]

24 adult Bergamasca × Massese sheep (47 ± 5 kg)	Bilateral lateral meniscectomy	Autologous abdominal adipose tissue.	The autologous abdominal adipose tissue was digested with 0.075% collagenase II for 1 h and then, the reaction was stopped with complete medium. Cells were centrifuged and 1 mL of suspension was injected into the joint. The total procedure took nearly 1 h and 30 min	N.A.	3 and 6 months	[21]

30 healthy adult male Sprague–Dawley rats (age: 8–10 weeks, weight: 300–350 g)	Sodium iodoacetate (3 mg/50 μl) was used to induce OA in the left knee joint of rats	Adipose tissue surrounding the rat kidney.	The adipose tissue was cut into small pieces and washed in PBS and was digested using 0.2% collagenase I (Sigma–Aldrich, St. Louis, MO, USA) at 37°C for 40 min with gentle shaking at 10-min intervals. The cell suspension was centrifuged at 700 × *g* for 10 min at 4°C. The supernatant was discarded, and the pellet was resuspended in PBS, and fltered through a 70-μm cell strainer (Corning Inc., Corning, NY, USA)	Each rat was intra-articularly injected with 5 × 10^6^ cells/50 μL of SVF.	14 days	[22]

All NOD.CB17-Prkdcscid/J male mice	Articular injury models were established by scrapping the knee cartilage on both hind legs using a very fine needle (32.5 G)	49 consenting patients.	Transpose RT System (InGeneron Inc., Houston, TX)	5 × 10^5^ cells/50 μl	30 and 60 days	[23]

## Data Availability

The data used to support the findings of this study are included within the article. The raw data that support the findings of this study are available on request from the corresponding author, upon reasonable request.
